# Comparison of Retinal Changes Following Silicone Oil and Perfluoropropane Gas Tamponade for Proliferative Diabetic Retinopathy Patients

**DOI:** 10.3389/fphys.2022.915563

**Published:** 2022-06-23

**Authors:** Tan Wang, Erqian Wang, Huan Chen, Ningning Li, Hanyi Min

**Affiliations:** ^1^ Department of Ophthalmology, Peking Union Medical College Hospital, Chinese Academy of Medical Sciences and Peking Union Medical College, Beijing, China; ^2^ Key Laboratory of Ocular Fundus Diseases, Chinese Academy of Medical Sciences and Peking Union Medical College, Beijing, China; ^3^ Operating Room, Peking Union Medical College Hospital, Chinese Academy of Medical Sciences and Peking Union Medical College, Beijing, China

**Keywords:** proliferative diabetic retinopathy, pars plana vitrectomy, retinal nerve fiber layer, silicone oil, perfluoropropane, optical coherence tomography, angiography

## Abstract

**Purpose:** To investigate the different tamponade effects of intravitreal silicone oil (SO) and perfluoropropane gas on the retinal structure and vasculature in proliferative diabetic retinopathy (PDR) patients.

**Methods:** Thirty-eight eligible patients (47 eyes) with PDR requiring pars plana vitrectomy (PPV) were enrolled in the prospective observational study. Subjects were divided into two groups after PPV: SO group subjects underwent SO tamponade, whereas Gas group subjects underwent perfluoropropane gas tamponade. The primary outcomes of this study were longitudinal changes in retinal structure and vasculature between 10 and 90 days after the operation. Secondary outcomes were longitudinal changes in peripapillary retinal nerve fiber layer (pRNFL) thickness between 10 and 90 days after the operation in each sector.

**Results:** Thirty-six eyes of 27 patients with a median age of 56.6 ± 9.8 years completed follow-up and were statistically analyzed. No significant difference in demographics or clinical characteristics was found between the two groups. Eyes in the SO group had a statistically significant decrease in pRNFL thickness at 90 days after PPV (*p* < 0.001), and there was a significant intergroup difference compared with the Gas group *(p* = 0.001), except for the temporal sector. Eyes in the Gas group had a statistically significant increase in parafoveal vessel density (VD) of the superficial vascular complex (SVC) at 90 days after PPV (*p* = 0.023), although there was no significant intergroup difference. The type of tamponade, changes in full retina thickness, and parafoveal SVC VD showed a significant correlation with changes in pRNFL thickness (all *p* < 0.05).

**Conclusion:** SO tamponade resulted in a significantly greater decrease in pRNFL over 90 days than gas tamponade in patients with PDR. In addition, the change in the pRNFL was significantly correlated with changes in full retina thickness and SVC VD after the operation.

## Introduction

Severe vision loss occurs commonly in patients with proliferative diabetic retinopathy (PDR) as a result of neovascularization and fibrovascular proliferation (Moss et al., 1998). Pars plana vitrectomy (PPV) is an indicated treatment for patients with PDR when nonclearing vitreous hemorrhage (VH), tractional retinal detachment (TRD), or macula-threatening retinal detachment occur. However, when patients with PDR undergo PPV with higher grades of vitreoretinal (VR) adhesion, VR adhesion removal during PPV is challenging and may result in posterior and/or peripheral retinal holes (Michels and De Bustros, 1988; Gupta et al., 2012). Vitreous substitutes of SO and long-acting gas have been reported to be useful during PPV in cases with advanced diabetic complications, complex rhegmatogenous retinal detachment, and proliferative vitreoretinopathy (Schwartz et al., 2014).

Although SO and long-acting gas tamponade are currently in wide use for patients with PDR and high-grade VR adhesion undergoing PPV, few studies have compared the effects of different tamponades on the postoperative retinal thickness and perfusion function of the individual retina in patients with PDR.

Advances in optical coherence tomography (OCT) technology enable the detection of macular architectural changes, while OCT angiography (OCTA) is able to precisely detect perfusion function, such as vessel density (VD) of the superficial vascular complex (SVC), deep vascular complex (DVC) on the macula, and radial peripapillary capillaries (RPC) (Spaide et al., 2018;Christou et al., 2021).

In this study, we evaluated such longitudinal changes in postoperative retinal thickness and perfusion function in patients with PDR who had undergone PPV treatments by comparing the differences in cohorts administered long-acting gas versus SO tamponades.

## Methods

This prospective, observational study was conducted at the Department of Ophthalmology, Peking Union Medical College Hospital (PUMCH). This study adhered to the tenets of the Declaration of Helsinki. The Institutional Review Board of PUMCH approved and authorized the patient consent forms and the study protocol in May 2018. Signed informed consent was obtained from all patients after they had been informed of the nature and possible consequences of the study procedures.

### Research Participants

Patients with PDR requiring single PPV following intravitreal SO or sterilized air (gas) tamponade between June 2018 and February 2019 were enrolled in the study. The study was not blinded to surgeons or patients or randomized to groups but was blinded to data analysts.

The inclusion criteria were as follows: 1) type I or II DM was diagnosed and medically managed; 2) patient aged over 18 years; 3) PDR with indications for PPV, including macula-threatening retinal detachment, TRD, and nonclearing VH; Snellen best-corrected visual acuity ranged from 20/40 to light perception with projection in the subject’s study eye.

The exclusion criteria were as follows: 1) high intraocular pressure (IOP) prior to PPV or high IOP for more than 1 week after PPV (>22 mmHg) under medicine; 2) a history of glaucoma, ischemic optic neuropathy, uveitis, or other retinal or optic nerve diseases; 3) a history of intraocular surgery except for refractive and cataract surgery; 4) a high myopia of over—6.00 diopters or axial length (AL) >26.50 mm; 5) opacity of the lens or cornea or opaque refractive media at any follow-up visit; and 6) medically uncontrolled systemic hypertension during the follow-up period (systolic. 200 mmHg or diastolic. 120 mmHg); 7) recurrent retinal detachment within 90 days after PPV, which required a secondary surgical repair; 8) SO removal was performed less than 90 days after PPV; 9) failure to complete follow-up or failure to follow-up on time; or 10) poor image quality: OCT/OCTA signal strength index < 6.

Subjects enrolled in the study were allocated to one of the following treatment cohorts: SO group participants underwent vitreous substitution with 1,000 centistoke SO during PPV, whereas the Gas group participants underwent vitreous substitution with 12% C3F8 gas during PPV. SO was chosen for patients who were traveling by airplane, had poor vision in their fellow eye, could not maintain a face-down position, or needed early good postoperative vision. SO was also chosen in some patients with inferior retinal detachment or avulsion.

### Evaluations and Surgical Techniques

Visual acuity (VA) with a Snellen chart, IOP measurement, biomicroscopy of the anterior and posterior segments, axial length, HbA1c and SD-OCT were measured or performed before PPV and 10 and 90 days after PPV. We also collected the following clinical characteristics: phakic eyes/pseudophakic eyes, duration of diabetes and type of diabetes, location and number of retinal breaks, whether hypertension was complicated and duration of hypertension.

Informed consent was obtained preoperatively. Before surgery, adequate doctor–patient communication was carried out with each of our patients. One surgeon (H.M.) performed procedures.

First, 0.05 mg of ranibizumab for pretreatment (Lucentis; Novartis) was injected into the vitreous cavity under topical anesthesia. One week later, a single PPV plus SO or 12% C3F8 gas tamponade was performed under retrobulbar anesthesia using the Constellation Vision System (Alcon Laboratories, Inc., Texas, United States) and a 25G trocar cannula system. The vitreous cavity was cleaned, and the peripheral vitreous was trimmed with an indentation. The proliferated membrane was peeled completely and/or separated into pieces until the retina could be attached completely. After fluid-gas exchange, PRP was performed.

SO (Oxane 5700, Bausch & Lomb, Rochester, N.Y., United States) or 12% C3F8 gas was injected fully until normal IOP was attained. While all sclerotomies were sutured in SO tamponade cases, only the leaking sclerotomies were sutured in gas tamponade cases. Following surgeries, patients used topical antibiotics, corticosteroids, and IOP-lowering medications as needed. The prone position or head-down position was adopted within 2 weeks in the two groups. IOP-lowering medications would be used only in cases of need.

No patient underwent combined cataract surgery during the procedure. The three follow-up visit times for all participants occurred at 10, 30, and 90 days after PPV. SO removal was performed approximately 3 months after SO tamponade surgery.

### OCT Imaging and Processing

Optic disk and macula evaluation were performed using the spectral domain OCT device RTVue XR Avanti (Optovue, Inc., Fremont, CA, United States). This device uses a light source of 840 nm and operates at a scan speed of 70,000 A-scans per second. Images on a 3 mm × 3 mm macula and a 4.5 mm × 4.5 mm optic disc scan were captured *via* dilated pupil capture after pupillary dilation using 1% tropicamide drops.

For quantitative assessment of the parafoveal VD of SVC, DVC, and RPC, we used the built-in software AngioVue AngioAnalytics (Version 2017.1, Optovue Inc., Fremont, CA, United States). This software utilizes a split-spectrum amplitude decorrelation angiography algorithm with a three-dimensional projection artifact removal technique. All image acquisitions were made by one investigator (E.W.). The OCT/OCTA signal strength index (from 1 to 10) was also provided by the software.

### Outcome Measures

The primary outcome measures of this study were longitudinal changes in retinal thickness and perfusion function between 10 and 90 days after PPV, including pRNFL thickness, RPC VD, inner limiting membrane to inner plexiform layer thickness, full retinal thickness, SVC VD, and DVC VD. Secondary outcome measures were longitudinal changes in pRNFL thickness between 10 and 90 days after PPV in each sector.

### Sample Size Calculation and Statistical Analysis

The longitudinal change in pRNFL thickness between 10 and 90 days after PPV in the SO group and Gas group was found to be −22.5 μm and −4.0 μm, respectively, by sampling. Assuming a study power of 90%, an alpha of 0.05, and equal group allocation, 12 subjects per cohort was determined to be the minimum sample size needed. Once 12 subjects per cohort completed a 90 day follow-up, enrollment ended, and the remaining subjects who were already enrolled were allowed to complete follow-up. Statistical analysis was performed using SPSS software (version 22.0; SPSS Inc., Chicago, IL). The normal distribution of all variables was verified by the Kolmogorov–Smirnov method. Baseline clinical characteristics were compared between the two groups (SO vs. Gas) using an independent Student’s *t* test, Mann–Whitney U test, and Fisher’s exact test. The paired *t* test or Wilcoxon signed rank test was used to compare the parameters between the two follow-up visits. The intergroup differences in longitudinal changes in evaluation indicators between 10 and 90 days after PPV were tested using an independent Student’s *t* test or Mann–Whitney U test. Correlations between the change in pRNFL thickness and other variables were evaluated using Spearman’s correlation analysis. A *p* value of less than 0.05 was considered statistically significant.

## Results

### Basic Information

**FIGURE 1:**
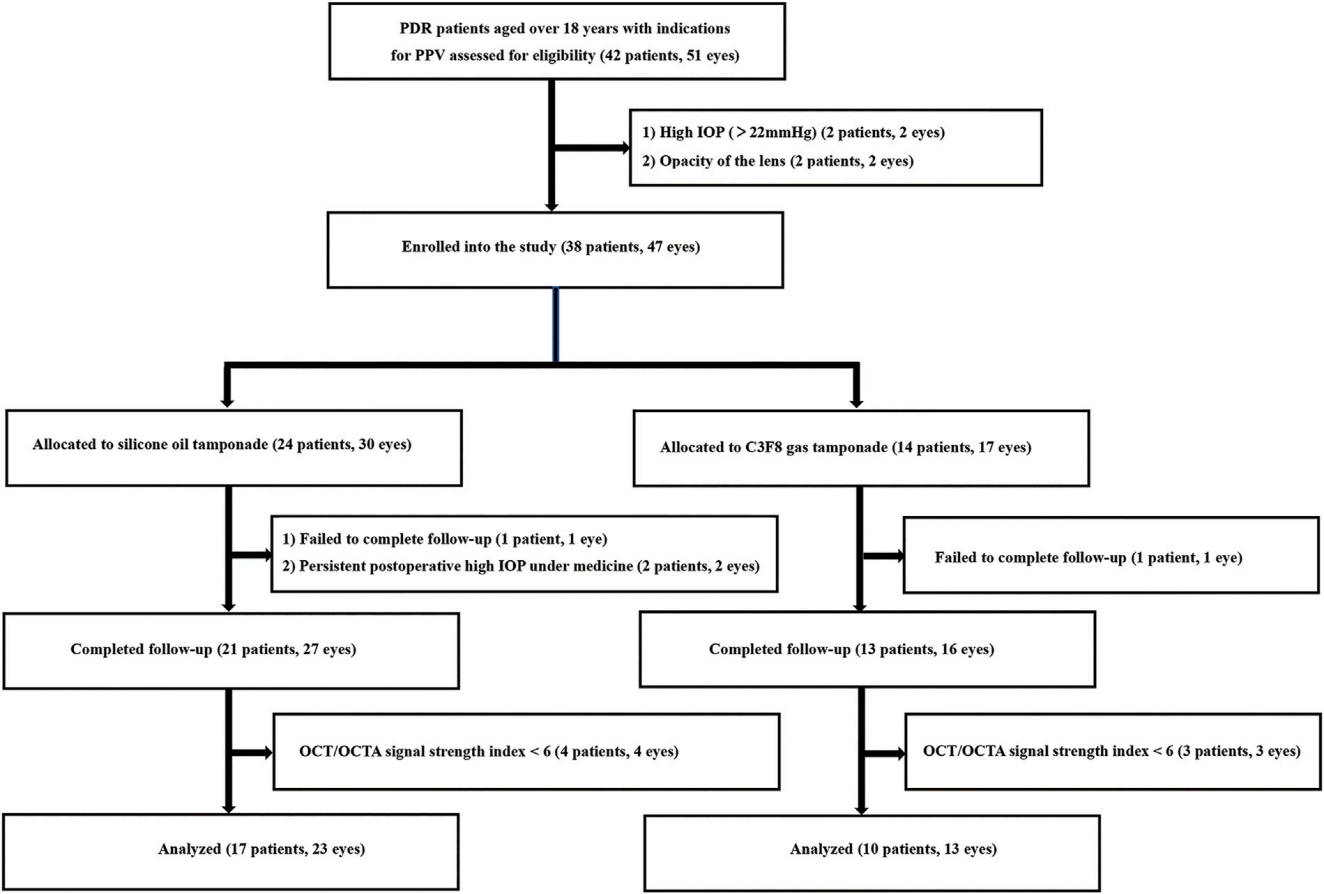
Study flowchart. PDR, proliferative diabetic retinopathy; PPV, pars plana vitrectomy; OCT, optical coherence tomography; OCTA, optical coherence tomography angiography; IOP, intraocular pressure; SO, silicone oil.

A total of 38 eligible patients (47 eyes) with PDR were enrolled and underwent a baseline examination and PPV. Among these, 11 patients (11 eyes) were excluded for the following reasons: failure to complete follow-up (2 patients, 2 eyes), postoperative persistent high IOP under medicine (2 patients, 2 eyes), and poor image quality (7 patients, 7 eyes; Figure 1). A total of 27 patients (36 eyes, 15 females and 12 males) completed all follow-up visits and were statistically analyzed. The median age of the patients was 56.6 ± 9.8 years. The mean time of DM was 11.9 ± 10.0 years. The mean axial length was 23.1 ± 0.8 mm. The median logMAR VA was 2.0 ± 1.7 (Snellen equivalent 20/2000) before the operation and 1.5 ± 1.0 (Snellen equivalent 20/632) 90 days after the operation (*p* = 0.125). The mean IOP was 14.8 ± 2.9 mmHg before the operation and 16.5 ± 5.1 mmHg 90 days after the operation (*p* = 0.066). Three eyes in the SO group and one eye in the gas group had elevated IOP not exceeding 30 mmHg in the first postoperative week and were controlled with medical therapy. As shown in Table 1, no significant difference in demographics or clinical characteristics was found between the two groups. The SO was kept well in the vitreous cavity in all patients in the SO group and never escaped to the anterior chamber during follow-up.

**TABLE 1:** Baseline characteristics of subjects included in the analysis^a^

Variables	SO group (n = 23)	Gas group (n = 13)	*p* ^†^
Age, y	56.0 ± 15.0	58.6 ± 7.0	0.478
Male gender (%)	9 (39.1)	6 (46.2)	0.736
Right Eye (number [%])	8(34.8)	6 (65.2)	0.723
Phakic eyes/pseudophakic eyes	17/3	13/0	0.261
Visual acuity (LogMAR)	2.0 ± 1.4	1.6 ± 0.9	0.102
Axial length (mm)	23.1 ± 0.6	23.0 ± 0.9	0.967
IOP (mmHg)	14.7 ± 3.0	15.2 ± 2.6	0.589
HbA1c (%)	7.6 ± 3.4	8.0 ± 1.4	0.619
Duration of diabetes (years)	7.5 ± 13.4	15.4 ± 7.4	0.061
Type 2 diabetes (number [%])	21 (91.3)	13 (100.0)	0.525
Hypertension (number [%])	12 (52.2)	8 (61.5)	0.731
Duration of hypertension (years)	2.0 ± 5.0	1.0 ± 11.0	0.733

a Quantitative data and qualitative data are expressed as the mean ± SD, or median ± IQR, and number of people (%), respectively.

^†^ *p* values refer to independent Student’s t test, Mann–Whitney U test and Fisher’s exact test were used to explore the differences in characteristics between two groups.

IOP, intraocular pressure.

### Longitudinal Changes in the Two Groups

Longitudinal changes in all evaluation indicators between 10 and 90 days after PPV in the two groups are presented in Table 2. Eyes in the SO group had a statistically significant decrease in pRNFL thickness at 90 days after PPV (10 days after PPV: 148.2 ± 56.3, 90 days after PPV: 102.6 ± 50.8 μm, *p* < 0.001). Eyes in the Gas group had a statistically significant increase in parafoveal SVC VD at 90 days after PPV (10 days after PPV: 34.4 ± 3.8%, 90 days after PPV: 36.6 ± 7.0%, *p* = 0.023).

**TABLE 2:** Longitudinal changes in evaluation indicators in the SO group and Gas group^a^

Groups	Measurements	10 days after PPV	90 days after PPV	*p* ^†^
SO Group	pRNFL thickness (μm)	147.0 ± 61.1	106.1 ± 49.8	<**0.001**
	RPC VD (%)	42.3 ± 6.1	42.2 ± 5.0	0.420
	ILM_IPL thickness (μm)	119.8 ± 50.7	125.4 ± 43.9	0.415
	Full retinal thickness (μm)	414.6 ± 99.1	379.5 ± 123.0	0.062
	SVC VD (%)	36.8 ± 5.2	35.2 ± 8.9	0.940
	DVC VD (%)	40.9 ± 6.4	40.0 ± 6.3	0.528
Gas Group	pRNFL thickness (μm)	111.2 ± 19.4	104.8 ± 15.7	0.235
	RPC VD (%)	42.8 ± 6.5	42.6 ± 2.9	0.311
	ILM_IPL thickness (μm)	112.0 ± 21.7	111.3 ± 22.4	0.972
	Full retinal thickness (μm)	377.2 ± 55.3	371.3 ± 64.1	0.531
	SVC VD (%)	34.4 ± 3.8	36.6 ± 7.0	**0.023**
	DVC VD (%)	41.7 ± 3.9	40.1 ± 4.8	0.321

Bold values indicate statistically significant differences.

a Quantitative data are expressed as the mean ± standard deviation or median ± interquartile range.

^†^ *p* values refer to the paired *t* test or Wilcoxon signed rank test exploring the differences in evaluation indicators between 10 and 90 days after PPV.

PPV, pars plana vitrectomy; SO, silicone oil; pRNFL, peripapillary retinal nerve fiber layer; RPC, radial peripapillary capillaries; VD, vessel density; ILM, inner limiting membrane; IPL, inner plexiform layer; SVC, superficial vascular complex; DVC, deep vascular complex.

### Different Tamponade Effects Between the Two Groups and Analysis of the Related Factors

Intergroup comparisons of all evaluation indicators for the longitudinal changes between 10 and 90 days after PPV are shown in Table 3. Compared to the Gas group, the SO group exhibited a greater decrease in pRNFL thickness, which led to a significant difference (SO: −26.3 ± 31.3 μm, Gas: −5.2 ± 17.3 μm, *p* = 0.001). No significant results were observed in other indicators of retinal thickness and capillary plexus flow density changes during follow-up.

**TABLE 3:** Intergroup differences in longitudinal changes in evaluation indicators^a^.

Measurements	Changes between 10 and 90 days after PPV		
	SO group	Gas group	*p* ^†^
pRNFL thickness (μm)	−21.9 ± 31.8	−5.1 ± 12.6	**0.002**
RPC VD (%)	0.5 ± 3.7	1.1 ± 3.2	0.895
ILM_IPL thickness (μm)	−5.7 ± 33.2	−0.7 ± 16.4	0.510
Full retinal thickness (μm)	−39.0 ± 95.2	−5.9 ± 33.0	0.141
SVC VD (%)	−0.1 ± 5.6	1.9 ± 2.9	0.164
DVC VD (%)	−0.9 ± 6.5	−1.5 ± 5.3	0.758

Bold values indicate statistically significant differences.

a Quantitative data are expressed as the mean ± standard deviation or median ± interquartile range.

^†^ *p* values refer to independent Student’s *t* test or the Mann–Whitney U test exploring the intergroup differences in longitudinal changes in evaluation indicators between 10 and 90 days after PPV.

PPV, pars plana vitrectomy; SO, silicone oil; pRNFL, peripapillary retinal nerve fiber layer; RPC, radial peripapillary capillaries; VD, vessel density; ILM, inner limiting membrane; IPL, inner plexiform layer; SVC, superficial vascular complex; DVC, deep vascular complex.

Table 4 shows the correlation between changes in pRNFL thickness and other variables. The type of tamponade, changes in full retina thickness and parafoveal SVC VD showed a significant correlation with changes in pRNFL thickness (*p* < 0.001, *p* = 0.024, and *p* = 0.042, respectively).

**TABLE 4:** Correlations between changes in pRNFL thickness and other variables.

Measurements	Changes of pRNFL thickness	
	Correlation coefficient	p*
Changes of RPC VD	0.166	0.334
Changes of ILM_IPL thickness	0.327	0.052
Changes of full retina thickness	0.375	**0.024**
Changes of SVC VD	0.341	**0.042**
Changes of DVC VD	0.017	0.921
Type of tamponade	0.523	**0.001**

Bold values indicate statistically significant differences.

*p values refer to Spearman's correlation analysis exploring the correlations between the changes in pRNFL, thickness and other variables.

pRNFL, peripapillary retinal nerve fiber layer; RPC, radial peripapillary capillaries; VD, vessel density; ILM, inner limiting membrane; IPL, inner plexiform layer; SVC, superficial vascular complex; DVC, deep vascular complex.

### Changes in pRNFL Thickness in Each Sector

**FIGURE 2:**
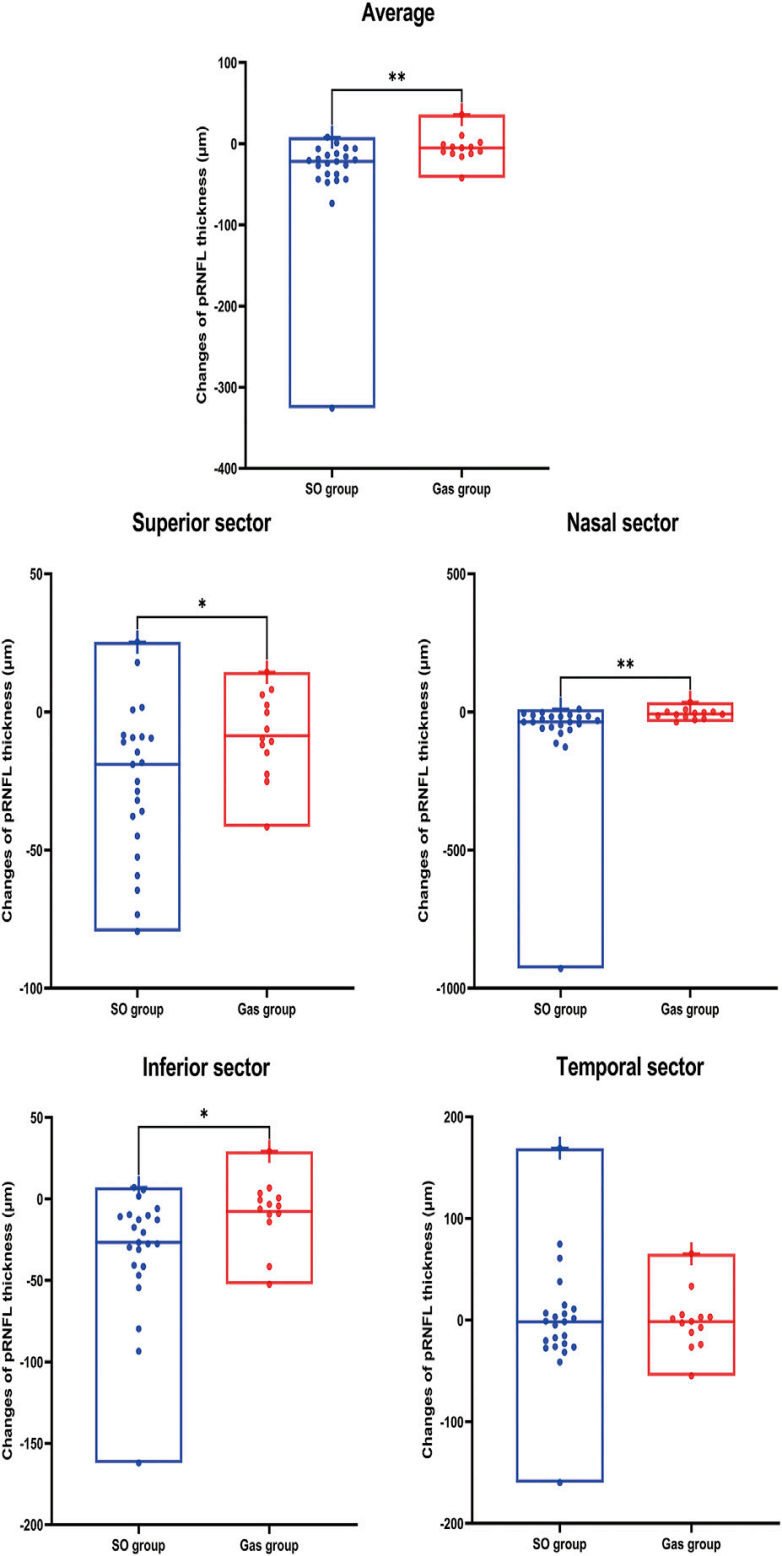
Changes in pRNFL thickness on average and in each sector were compared between the two groups. SO, silicone oil. *p* value: *, < 0.05; **, < 0.01; ***, < 0.001; ****, < 0.0001.

As shown in Table 5 and Figure 2, Superior (*p* = 0.001), Nasal (*p* < 0.001), and Inferior *p* < 0.001) pRNFL thicknesses at 90 days after PPV significantly decreased compared with those at 10 days after PPV in the SO group. In addition, the decreases in pRNFL thickness in the superior (*p* = 0.024), nasal (*p* = 0.003), and inferior (*p* = 0.011) sectors in the SO group were significantly greater than those in the Gas group.

**TABLE 5:** Intergroup differences in longitudinal changes in peripapillary retinal nerve fiber layer thickness in each sector^a^.

Measurements	SO group at 10 days after PPV	SO group at 90 days after PPV	*p* ^†^	Gas group at 10 days after PPV	Gas group at 90 days after PPV	*p* ^†^	Changes (between10 days and 90 days after PPV in SO group)	Changes (between10 days and 90 days after PPV in gas group)	*p* ^‡^ (intergroup difference of changes between 10 and 90 days after PPV)
Superior	147.3 ± 76.5	122.1 ± 79.2	**0.001**	132.3 ± 19.3	123.7 ± 21.7	0.067	−25.5 ± 27.5	−8.6 ± 15.4	**0.024**
Nasal	108.5 ± 79.7	79.8 ± 27.5	<**0.001**	88.0 ± 25.7	74.7 ± 16.7	0.055	−36.2 ± 43.9	−8.5 ± 21.2	**0.003**
Inferior	178.0 ± 76.2	145.5 ± 59.6	<**0.001**	136.9 ± 24.0	129.1 ± 26.4	0.198	−26.7 ± 31.1	−4.3 ± 13.8	**0.011**
Temporal	122.7 ± 80.3	130.2 ± 68.6	0.563	103.7 ± 37.6	100.1 ± 34.8	0.701	−1.57 ± 37.2	−1.3 ± 22.1	0.974

Bold values indicate statistically significant differences.

a Quantitative data are expressed as the mean ± standard deviation or median ± interquartile range;

^†^ *p* values referring to the paired *t* test or Wilcoxon signed rank test were used to compare the parameters between the two follow-up visits;

^‡^ *p* values referring to independent Student’s t test or Mann–Whitney U test were used to explore the intergroup differences in longitudinal changes in evaluation indicators between 10 and 90 days after PPV.

PPV, pars plana vitrectomy; SO, silicone oil.

## Discussion

SO is frequently used as a vitreous substitute during PPV surgery in patients with PDR (Castellarin et al., 2003; Altan et al., 2008). It is able to reduce the incidence of NVG, act as a barrier to angiogenic factors, and allow further photocoagulation in the early postoperative period when endolaser uptake is inadequate (McLeod, 1986; Pearson et al., 1993; Castellarin et al., 2003; Morphis et al., 2012). However, SO tamponade has difficulty acquiring a complete oil fill during PPV and requires a second surgery for extraction from the eye. Many surgeons prefer using C3F8 gas as a vitreous substitute in PDR patients due to its characteristics of higher surface tension, buoyant force, and spontaneous reabsorption compared with that of SO (Castellarin et al., 2003;Schwartz et al., 2014).

VR surgeons frequently confront a dilemma when determining which vitreous substitute to select during PPV in advanced indications of PDR, such as TRD or extensive fibrous proliferation, especially when iatrogenic retinal breaks occur. To avoid postoperative problems such as proliferative vitreoretinopathy, retinal detachment, or VH, surgeons must take precautions before performing the procedure.

Vitreous substitution of BSS, air, gas, and SO tamponade has been compared by several retrospective studies without reporting a difference in postoperative outcomes (Yorston et al., 2008;Ostri et al., 2014;Dikopf et al., 2015;Jackson et al., 2016). A randomized clinical trial (RCT) that compared gas and SO tamponade in eyes with diabetic retinopathy showed that the postoperative BCVA was poorer in SO tamponade eyes than in gas tamponade eyes at 6 months (Rush et al., 2021). In addition, some studies have observed a decline in VA following SO tamponade in patients undergoing PPV surgery for diabetic TRD, and SO has been hypothesized to be harmful to the retina (Yorston et al., 2008;Jackson et al., 2016). Pallor of the optic nerve was more prevalent following SO tamponade, implying that long-term SO tamponade may contribute to optic nerve injury (Shroff et al., 2018).

There is a paucity of studies investigating detailed changes in the retina following SO or gas tamponade in PDR patients undergoing PPV surgery. However, some studies reported changes in eyes with rhegmatogenous retinal detachment (RRD). Zhou et al. (2020) reported that compared to gas, SO could have more negative tamponade effects on both fundus vasculature and structure. A retrospective study completed by Inan et al. (2020) found thinning in the layers of the ganglion cell layer, outer plexiform layer, and outer nuclear layer in the SO group. Moreover, Liu et al. (2021) reported that the parafoveal vessel densities in the SVC and the corresponding inner retinal thickness were significantly reduced in the affected eyes compared to the contralateral eyes in the SO group but similar between the affected eyes and the contralateral eyes in the Gas group. These studies suggest that the use of SO tamponade affects retinal layer thickness more significantly than perfluoropropane gas tamponade in eyes with RRD.

In this study, we investigated the effects of SO and gas on the retina in eyes with PDR by comparing changes in postoperative retinal thickness and VD using SD-OCT *in vivo*. We found that SO tamponade had a significant effect on the postoperative decrease in pRNFL thickness, and there was a significant intergroup difference in the changes between 10 and 90 days after PPV surgery. To further explore the factors associated with changes in the pRNFL after PPV, we found that the type of tamponade (SO or C3F8 Gas) had a strong correlation with changes in pRNFL thickness in patients with PDR. These findings strongly support the appearance of optic nerve pallor observed in an earlier study (Shroff et al., 2018). Zhou et al. (2020) also reported that compared to gas tamponade, SO tamponade resulted in a more pronounced decrease in nerve fiber layer thickness, although the study was carried out in eyes with RRD.

However, the exact mechanism by which SO significantly reduces the thickness of pRNFLs remains uncertain. First, SO-induced mechanical stress in the fovea can result in the subsequent early loss of ONL cell bodies (Dooley et al., 2015). Although mechanical stress may be related to increased IOP, in our study, eyes with IOP elevation could immediately be controlled with IOP-lowering agents, and none of the eyes showed IOP elevation greater than 30 mmHg. Severe optic neuropathy could result from SO tamponade through subretinal migration of SO (Knecht et al., 2007;Majid et al., 2008). Migration of phagocytosed emulsified oil bubbles by macrophages might be a mechanism for subretinal migration of SO (Budde et al., 2001). However, subretinal migration of SO was not found in our study. Another possible mechanism of retinal thinning is SO-related idiopathic reactions or retinal ionic environmental changes (Winter et al., 2000). Local changes in concentrations of potassium due to the failure of potassium siphoning by Müller cells and apoptosis and subsequent neuronal degeneration changes could be indirect mechanisms (Winter et al., 2000; Newsom et al., 2004). It was reported that the micromolecule in SO might diffuse from the oil and penetrate into retinal tissue, inducing inflammation and retinal toxicity (Gonvers et al., 1986; Gabel et al., 1987). This seems to be consistent with our analysis, which showed that changes in full retinal thickness and SVC VD in the macula had a strong correlation with changes in pRNFL thickness.

It is somewhat surprising that no significant intergroup differences were found in the temporal sector. A possible explanation for this might be that the temporal side is closer to the macula and has a relatively good blood supply. The significant correlation observed in the correlation analysis in this study between changes in pRNFL thickness and SVC VD in the macula might be an explanation.

Furthermore, changes in perfusion function between 10 and 90 days after PPV surgery were also investigated after PPV. It was interesting that gas tamponade had a significant effect on the postoperative increase in parafoveal SVC VD, while SO tamponade had a mild effect on the postoperative decrease in parafoveal SVC VD. However, there was not a significant intergroup difference. This result was similar to that found in a study conducted in patients with primary RRD (Zhou et al., 2020).

Regarding the effects on full retinal thickness and thickness between the inner limiting membrane and inner plexiform layer, SO had more negative tamponade effects than Gas, with no statistically significant intergroup differences. These trends were almost consistent with previous studies in RRD eyes (Inan et al., 2020; Zhou et al., 2020; Liu et al., 2021), although the etiology and mechanisms of RRD and PDR were quite different.

This study has some limitations. First, the two vitreous substitution groups in this prospective, observational study were not randomized but rather based on patient need or ocular condition because randomization would raise some ethical issues. Second, on account of the tamponade choice and timely SO removal in clinical practice in our ophthalmic center, the observation time was relatively short, and only comparisons between gas and SO tamponade were analyzed. Extended SO tamponade, on the other hand, may be followed by more adverse effects, such as glaucoma, cataract formation, and peri-SO reproliferation (Tsui et al., 2020). Furthermore, the sample size of our study was relatively small. In the future, large-scale clinical trials with other vitreous substitution groups, such as BSS, air, or short-acting gas, are needed.

In conclusion, SO tamponade had a significantly greater decrease in pRNFL over 90 days than perfluoropropane gas tamponade, whereas no significant intergroup difference was observed in the temporal sector in patients with PDR. The changes in the pRNFL were correlated with changes in full retina thickness and parafoveal SVC VD after the operation. Therefore, surgeons should be aware of the reduction of pRNFL thickness in patients with SO tamponade to manage them appropriately regarding any changes in retina.

## Data Availability

The raw data supporting the conclusion of this article will be made available by the authors, without undue reservation.
